# Supplementary material for: In Vitro Regeneration from Leaf Explants of Helianthus verticillatus, a Critically Endangered Sunflower
Source: Plants (Basel). 2024 Jan 18;13(2):285. doi: 10.3390/plants13020285 (PMC10820345; doi:10.3390/plants13020285)
Supplement: Supplementary file 1 [file plants-13-00285-s001.zip › Nowakowska Figure S1.pdf]

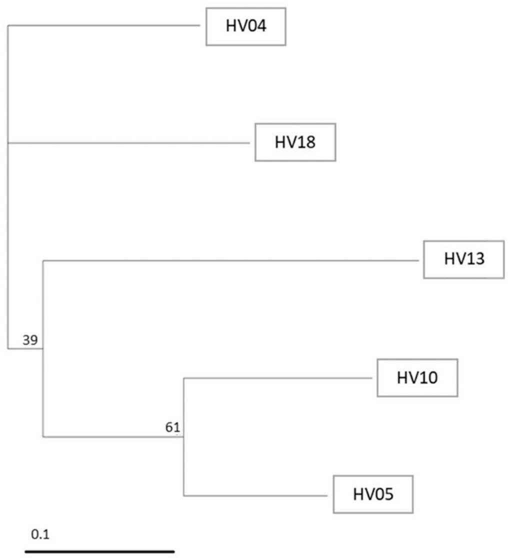

Figure S1: A dendrogram generated for genotypic data from 15 polymorphic microsatellite markers (SSRs), illustrating the genetic distance among the donor plants of *Helianthus verticillatus*. The matrix of genetic distances was calculated in R version 3.6.1 using RStudio version 1.2.5019, and the package *poppr* version 2.8.3 [68] with Bruvo's genetic distance [69] that regards the repeated motif lengths. Bootstrap support values for each split were calculated over 10,000 permutations of the dataset; only values ≥ 30 are displayed
