# Supplementary material for: In Vitro Regeneration from Leaf Explants of Helianthus verticillatus, a Critically Endangered Sunflower
Source: Plants (Basel). 2024 Jan 18;13(2):285. doi: 10.3390/plants13020285 (PMC10820345; doi:10.3390/plants13020285)
Supplement: Supplementary file 1 [file plants-13-00285-s001.zip › Nowakowska Table S1.pdf]

Table S1. Analysis of Variance (ANOVA) for the parameters examined for *in vitro* shoot regeneration rate from leaf explants of *Helianthus verticillatus*.

Tested Parameters	Variation Source	Sum of Squares	F Value	P value [Pr(>F)]
	Induction Medium (M)	273641.0	1709.1	<2.2$^{E-16***}$
Explants forming callus (%)	Genotype (G)	107.0	3.0	5.4^{E-02ns}
	M × G	1015.0	3.2	1.6$^{E-04***}$
	Induction Medium (M)	6598.3	24.9	<2.2$^{E-16***}$
Explants forming shoots (%)	Genotype (G)	2922.3	49.6	3.0$^{E-15***}$
	M × G	6830.7	12.9	<2.2$^{E-16***}$
	Induction Medium (M)	3.8	10.3	9.9$^{E-11***}$
Number of shoots per explants	Genotype (G)	1.6	19.7	7.9$^{E-08***}$
	M × G	4.5	6.0	2.8$^{E-09***}$

*—significant at $p < 0.05$; **—significant at $p < 0.01$; ***—significant at $p < 0.001$; ^{ns}—not significant.
