# Supplementary material for: In Vitro Regeneration from Leaf Explants of Helianthus verticillatus, a Critically Endangered Sunflower
Source: Plants (Basel). 2024 Jan 18;13(2):285. doi: 10.3390/plants13020285 (PMC10820345; doi:10.3390/plants13020285)
Supplement: Supplementary file 1 [file plants-13-00285-s001.zip › Nowakowska Table S2.pdf]

Table S2. Effect of explant source, induction medium, and genotype on the *in vitro* shoot regeneration rate of leaf explants from *Helianthus verticillatus*.

Explant Source	Induction Medium*	Genotype				
		HV04	HV05	HV10	HV13	HV18
		Explants forming callus (%)				
In vivo	MS1	98.2±5.1	100.0	100.0	97.2±7.8	98.2±5.1
	MS1CH	96.4±6.6	98.6±3.9	100.0	97.2±7.8	94.6±10.6
	MS3	100.0	98.6±3.9	100.0	100.0	100.0
In vitro	MS1	100.0	100.0	100.0	100.0	100.0
	MS1CH	100.0	100.0	100.0	100.0	100.0
	MS3	100.0	100.0	100.0	100.0	97.9±5.9
		Explants forming shoots (%)				
In vivo	MS1	0.0	29.2±23.0	2.8±7.8	54.2±22.6	16.7±14.4
	MS1CH	0.0	29.1±16.7	1.4±3.9	55.6±22.2	10.9±12.3
	MS3	0.0	19.4±22.8	26.4±5.7	47.2±16.6	17.9±10.1
In vitro	MS1	0.0	39.6±19.8	18.8±18.8	73.4±26.3	37.5±26.3
	MS1CH	0.0	39.6±17.7	20.9±21.4	75.0±17.7	20.8±14.8
	MS3	0.0	2.1±5.9	37.5±30.5	67.2±29.1	6.3±12.4
		Mean No. of shoots per explant				
In vivo	MS1	0.0	1.00±1.26	0.03±0.07	2.00±1.04	0.41±0.40
	MS1CH	0.0	0.71±0.53	0.03±0.07	2.25±1.45	0.25±0.28
	MS3	0.0	0.55±1.24	0.49±0.24	1.24±0.74	0.68±0.68
In vitro	MS1	0.0	1.15±0.43	0.63±1.06	3.75±1.86	1.23±1.13
	MS1CH	0.0	1.17±0.67	0.77±0.71	6.43±3.66	0.33±0.27
	MS3	0.0	0.02±0.06	0.96±1.01	4.69±1.45	0.27±0.55

For each genotype, data are the average (±SD) of 64 explants.

*MS medium supplemented with: 8.88 μM BA and 1.08 μM NAA (MS1); 8.88 μM BA, 1.08 μM NAA, and 500 mg × L^{-1} CH (MS1CH); 2.20 μM BA and 2.68 μM NAA (MS3)

For each evaluated trait, a three-way ANOVA was used to analyze the impact of factors (genotype, plant source, and induction medium) on shoot regeneration rate of leaf explants from *H. verticillatus* (Table 2). The triple interaction 'genotype × plant source × induction medium' was not significant for either of the traits. Subsequently, two-way ANOVAs were conducted to test all possible pairwise interactions due to the significant interactions between various factors (Figures 2a,b - 3ab; Table S3).
