# Supplementary material for: In Vitro Regeneration from Leaf Explants of Helianthus verticillatus, a Critically Endangered Sunflower
Source: Plants (Basel). 2024 Jan 18;13(2):285. doi: 10.3390/plants13020285 (PMC10820345; doi:10.3390/plants13020285)
Supplement: Supplementary file 1 [file plants-13-00285-s001.zip › Nowakowska Table S3.pdf]

Table S3. Two-way ANOVA analysis of the significance of pairwise interactions of among: *Helianthus verticillatus* genotype, explant source, and induction medium on plant regeneration frequency and the number of explants producing shoot.

**Tested parameters**	**Variation source**	**Sum of Squares**	**F value**	**P value [Pr(>F)]**
	Genotype × Induction Medium			
Explants forming shoots (%)	Genotype	100694.0	78.3	$<2.20^{E-16***}$
	Induction Medium	941.0	1.5	2.34^{E-01ns}
	Genotype × Induction Medium	12242.0	4.7	$2.05^{E-05***}$
Number of shoots per explants	Genotype	348.7	54.2	$<2.20^{E-16***}$
	Induction Medium	3.7	1.2	$3.16^{E-01***}$
	Genotype × Induction Medium	27.2	2.1	3.56^{E-02*}
	Genotype × Plant Source			
Explants forming shoots (%)	Genotype	100694.0	74.7	$<2.20^{E-16***}$
	Plant Source	4358.0	12.9	$3.97^{E-04***}$
	Genotype × Plant Source	3624.0	2.7	$3.21^{E-02***}$
Number of shoots per explants	Genotype	348.7	74.0	$<2.20^{E-16***}$
	Plant Source	36.9	31.3	$6.22^{E-08***}$
	Genotype × Plant Source	85.1	18.1	6.50^{E-03*}
	Induction Medium × Plant Source			
Explants forming shoots (%)	Induction Medium	941.0	0.6	5.41^{E-01ns}
	Plant Source	4358.0	5.7	1.78^{E-02*}
	Induction Medium × Plant Source	1989.0	1.3	2.74^{E-01ns}
Number of shoots per explants	Induction Medium	3.7	0.6	5.36^{E-01ns}
	Plant Source	36.9	12.4	$5.25^{E-04***}$
	Induction Medium × Plant Source	2.9	0.5	6.18^{E-01ns}

*Significant at $p<0.05$; **significant at $p<0.01$; ***significant at $p<0.001$; ^{ns} not significant.

Due to lack of significance for the triple interaction "genotype × plant source × induction medium" on plant regeneration frequency and the number of explants producing shoot, all possible pairwise interactions of studied factors (genotype, plant source, induction medium) were investigated separately using two-way ANOVAs, followed by post-hoc Tukey HSD tests ($\alpha = 0.05$).
