# Supplementary material for: In Vitro Regeneration from Leaf Explants of Helianthus verticillatus, a Critically Endangered Sunflower
Source: Plants (Basel). 2024 Jan 18;13(2):285. doi: 10.3390/plants13020285 (PMC10820345; doi:10.3390/plants13020285)
Supplement: Supplementary file 1 [file plants-13-00285-s001.zip › Nowakowska Table S4.pdf]

Table S4. Microsatellite loci used to examine genetic distance among the donor plants (HV04; HV05; HV10; HV13; HV18) of *Helianthus verticillatus*.

Locus	Forward (F) and Reverse (R) Primers 5′-3′	Repeat Motif	Number of Alleles	Allele Size Range (bp)
HV006[a]	F: CATGGGTGATCAATGGAGTG<br>R: CGGCACATAACAAGTGCTTC	$(gtga)_3$	2	227–267
HV017[a]	F: ACTTACCGTTGCATTTGGTG<br>R: GCTTATCCCTAGAACACGATTACAG	$(taa)_4$	2	109–112
HV021[a]	F: AAGTCGCACAGGAAGGAGATAG<br>R: TTGCTTGGAAGCCAATGAG	(ttgt)3	2	101-105
HV037[a]	F: GGTTAGGGTGAGGGTGGTG<br>R: AAGCCATAGTAAGTTCCTCTTACAAAC	$(tgca)_3$	3	155–179
HV042[a]	F: GGTTACAACGGTGGAAGTCG<br>R: TCCGGTTCACCAATTCATTC	$(ggc)_4$	2	367–373
Hvert002[b]	F: ATTCAGGGTTTCTGCCAGTGG<br>R: GAACTGATACCGACGCAAACC	$(ca)_2$	3	234-258
Hvert005[b]	F: GTCTTCAACTCAAACCACTAGCC<br>R: CAAGTTGTGCCCGATTTGTAGG	$(ag)_7$	2	215-233
Hvert015[b]	F: GGTTCTTACAACTCGAGGTGG<br>R: AACATCCAGTTGGGCTAACC	$(ta)_9$	3	155-185
Hvert026[b]	F: TTCCAGAGGCCGACTTCAAGG<br>R: ATAGGTGTGTGGAAGCCATGG	$(gaa)_6$	2	237-249
Hvert028[b]	F: GCGGATGATTCTTAGCACTAACC<br>R: TGGAAATAGAAAGAATTCCGGGC	$(cca)_6$	2	244-280
Hvert030[b]	F: CAACCATGCTGTCATCACTGG<br>R: GAGAATGGTGGTGTCCAGACC	$(tcc)_6$	3	170-179
Hvert033[b]	F: GTGGGTGAAAGGAAAGTGTTGG<br>R: GATCTCTCCATCTCTCGGTGC	$(gaa)_7$	2	130-136
Hvert042[b]	F: TTGGAAACAGCCTCTCTATCG<br>R: CGTCGCCATTAGAGACTTTGG	$(gttt)_7$	2	200-208
Hvert045[b]	F: ATGTTCCCTGCTCTTCGATCG<br>R: TTAATCCGCCCTGCAGTTAGG	$(catt)_5$	2	133-145
Hvert046[b]	F: ATGAGAAACCAGCTGTCGTCC<br>R: ACCCTCAAATCTCTTCTAAACC	$(agaa)_5$	3	282-318

[a] Loci renamed by Edwards et al. [65] and taken from Pashley et al. [64];

[b] Loci obtained from our transcriptome experiments (The reads were deposited in the Sequence Read Archive (SRA) database of the U.S. National Center for Biotechnology Information (NCBI) -PRNJA778959).
