# Supplementary material for: In Vitro Regeneration from Leaf Explants of Helianthus verticillatus, a Critically Endangered Sunflower
Source: Plants (Basel). 2024 Jan 18;13(2):285. doi: 10.3390/plants13020285 (PMC10820345; doi:10.3390/plants13020285)
Supplement: Supplementary file 1 [file plants-13-00285-s001.zip › Nowakowska Table S5.pdf]

Table S5. Media tested for *in vitro* shoot induction from leaf explants of *Helianthus verticillatus*, including plant growth regulators composition and their respective concentrations.

**Induction Medium**	**Plant growth regulators (µM)**	**References**
MS0	Without plant growth regulators	-
MS1*	BA (8.88) + NAA (1.08)	[41]
MS1CH*	BA (8.88) + NAA (1.08) + CH (500 µM × L^{-1})	[41]
MS2	BA (4.44) + NAA (0.54)	[42]
MS3 (KR)*	BA (2.20) + NAA (2.68)	[37]
MS4 (MSD4)	BA (2.20) + NAA (0.27)	[39]
MS5	ZEA^{RIB} (2.82)	[32]
MS6	BA (2.22)	[42]
MS7	BA (4.44)	[42]
MS8	BA (8.88)	[42]

*Media were used for further research in the second experiment that assessed the regeneration efficiency of different *H. verticillatus* genotypes, both in an *in vivo* and *in vitro* propagated plant
